# Two new species of *Alseodaphnopsis* (Lauraceae) from southwestern China and northern Myanmar: evidence from morphological and molecular analyses

**DOI:** 10.3897/phytokeys.138.38569

**Published:** 2020-01-10

**Authors:** Lang Li, Yun-Hong Tan, Hong-Hu Meng, Hui Ma, Jie Li

**Affiliations:** 1 Center for Integrative Conservation, Xishuangbanna Tropical Botanical Garden, Chinese Academy of Sciences, Mengla 666303, China Xishuangbanna Tropical Botanical Garden, Chinese Academy of Sciences Mengla China; 2 Center of Conservation Biology, Core Botanical Gardens, Chinese Academy of Sciences, Mengla 666303, China Core Botanical Gardens, Chinese Academy of Sciences Mengla China; 3 Southeast Asia Biodiversity Research Institute, Chinese Academy of Sciences, Yezin, Nay Pyi Taw 05282, Myanmar Southeast Asia Biodiversity Research Institute, Chinese Academy of Sciences Nay Pyi Taw Myanmar; 4 CAS-Key Laboratory of Tropical Forest Ecology, Xishuangbanna Tropical Botanical Garden, Chinese Academy of Sciences, Mengla 666303, China Xishuangbanna Tropical Botanical Garden Mengla China

**Keywords:** Kachin state, phylogenetic analysis, taxonomy, tropical montane forest, Yunnan province

## Abstract

*Alseodaphnopsis maguanensis* and *A. putaoensis*, two new species of *Alseodaphnopsis* (Lauraceae) from southwestern China (Yunnan Province) and northern Myanmar (Kachin State), are here described and illustrated based on both morphological and molecular evidence. They are morphologically similar to *Alseodaphnopsis rugosa* and phylogenetically closely related to *A. rugosa* and *A. hainanensis* respectively. Their preliminary conservation status is also estimated according to the IUCN Red List Categories and Criteria.

## Introduction

*Alseodaphnopsis* H. W. Li & J. Li, including nine species at present, is a recently described new genus of the Lauraceae (Mo et al. 2017a). Mo et al. (2017a) separated *Alseodaphnopsis* from the traditionally recognized tropical Asian genus *Alseodaphne* Nees based on both morphological and molecular evidence. The combination of principal morphological characters to distinguish the two genera (*Alseodaphnopsis* vs. *Alseodaphne*) includes: 1) twigs thick, 4–11 mm in diameter, not obviously whitish in color vs. thin, 2.5–4.5 mm in diameter, obviously whitish in color; 2) terminal buds perulate vs. not perulate; 3) perianth lobes persistent at least in young fruit vs. early deciduous; 4) inflorescences relatively large, 8.5–35 cm long, generally many-flowered, with 3–4 order of branching vs. 3–20 cm long, few-flowered, with 1–2 orders of branching; and 5) mature fruit relatively large, 3–5 cm vs. *<* 2.5cm in diameter (Mo et al. 2017a). In addition, *Alseodaphnopsis* species are distributed in the northern marginal zone of Asian tropics in southwestern China (also in Hainan island) and northern Vietnam while *Alseodaphne* species are mostly found in the tropics of south and southeast Asia (Kostermans 1973; Li et al. 2008; Mo et al. 2017a).

During recent field surveys in southwestern China (Maguan, Yunnan Province) and northern Myanmar (Putao, Kachin State), two unknown Lauraceae species were collected. Based on both morphological and molecular evidence, they were confirmed as new species of *Alseodaphnopsis* and closely related to *Alseodaphnopsis rugosa* (Merr. & Chun) H. W. Li & J. Li and *A. hainanensis* (Merr.) H. W. Li & J. Li respectively. In the work of Mo et al. (2017b), the specimens collected in Maguan (Yunnan Province, China) were treated as synonyms of *Alseodaphnopsis rugosa*. In this paper, these new *Alseodaphnopsis* species are described and illustrated.

## Material and methods

### Morphological studies

Morphological characters of the two new *Alseodaphnopsis* species were examined in detail based on dried specimens and fresh materials in field observations and compared with possible relatives based on the specimens from the HITBC, IBK, IBSC and KUN herbaria as well as images of specimens available on JSTOR Global Plants (http://plants.jstor.org/).

### Molecular studies and phylogenetic analyses

Total genomic DNA was extracted from silica-gel dried leaf material using the Plant Genomic DNA Kit (Tiangen, Beijing, China). Two nuclear DNA fragments, internal transcribed *spacer* (ITS) and the second intron of *LEAFY* gene (*LEAFY* intron II), were amplified and sequenced following the work of Li et al. (2011). ITS and *LEAFY* intron II sequences of other possibly related species involved here were obtained from GenBank according to the works of Li et al. (2011) and Mo et al. (2017a). Species examined in this study, voucher information, collection locality and GenBank accession numbers for ITS and *LEAFY* sequences are given in Table 1.

**Table 1.** Species examined in this study, voucher information, collection locality and GenBank accession numbers for ITS and *LEAFY* sequences.

Taxon	Voucher	Locality	ITS	*LEAFY*
**Ingroups**				
***Alseodaphne* (4)**				
*A. gigaphylla* Kosterm.	Arifiani DA657 (BO)	Indonesia, Java	HQ697181	HQ697004
*A. gracilis* Kosterm.	Li L. 20070187 (HITBC)	China, Yunnan	HQ697187	HQ697036
*A. huanglianshanensis* H. W. Li & Y. M. Shui	Li L. 20080006 (HITBC)	China, Yunnan	HQ697182	HQ697007
*A. semecarpifolia* Nees	Arifiani DA658 (BO)	Indonesia, Java	HQ697184	HQ697015
***Alseodaphnopsis* (8)**				
*A. andersonii* (King ex Hook. f.) Kosterm.	Li J. & Li L. 20070074 (HITBC)	China, Yunnan	FM957793	HQ697002
*A. hainanensis* Merr.	Li L. & Wang Z. H. JFL07 (HITBC)	China, Hainan	MG188587	MG188634
	Li L. & Wang Z. H. LMS10 (HITBC)	China, Hainan	MG188586	MG188633
*A. maguanensis* L. Li & J. Li	Li L. et al. GLQ45 (HITBC)	China, Yunnan	MN906900	MN906896
	Li L. et al. GLQ46 (HITBC)	China, Yunnan	MN906901	MN906897
*A. petiolaris* (Meissn.) Hook. f.	Chen J. Q. 07003 (HITBC)	China, Yunnan	FM957796	HQ697008
*A. putaoensis* L. Li, Y. H. Tan & J. Li	Li L. & Ma H. MM254 (HITBC)	Myanmar, *Kachin*	MN906902	MN906898
	Li L. & Ma H. MM266 (HITBC)	Myanmar, *Kachin*	MN906903	MN906899
*A. rugosa* Merr. & Chun	Li L. & Wang Z. H. MYH02 (HITBC)	China, Hainan	MG188585	MG188635
	Li L. & Wang Z. H. MYH08 (HITBC)	China, Hainan	MG188584	MG188640
*A. sichourensis* H. W. Li	Song Y. 33225 (HITBC)	China, Yunnan	MG188597	MG188626
*A. ximengensis* H.W. Li & J. Li	Li J. W. 1235 (HITBC)	China, Yunnan	MG188591	MG188599
***Dehaasia* (1)**				
*D. hainanensis* Kosterm.	Li L. & Wang Z. H. 20070373 (HITBC)	China, Hainan	FJ719308	HQ697026
***Machilus* (8)**				
*M. duthiei* King ex Hook. f.	Zhong J. S. 2006094 (HITBC)	China, Yunnan	FJ755425	HQ697055
*M. gongshanensis* H. W. Li	Chen J. Q. 07002 (HITBC)	China, Yunnan	FJ755416	HQ697047
*M. grijsii* Hance	Chen J. Q. et al. 2006028 (HITBC)	China, Guangdong	FJ755420	HQ697049
*M. kwangtungensis* Yang	Chen J. Q. et al. 2006027 (HITBC)	China, Guangdong	FJ755424	HQ697051
*M. monticola* S. Lee	Li L. & Wang Z. H. 20070323 (HITBC)	China, Hainan	FJ755418	HQ697057
*M. platycarpa* Chun	Chen J. Q. et al. 2006073 (HITBC)	China, Guangdong	FJ755421	HQ697067
*M. robusta* W. W. Sm.	Li J. 2002116 (HITBC)	China, Guangxi	FJ755426	HQ697071
*M. yunnanensis* Lec.	Zhong J. S. 2006093 (HITBC)	China, Yunnan	FJ755415	HQ697084
***Nothaphoebe* (1)**				
*N. umbelliflora* (Blume) Blume	Arifiani DA495 (BO)	Indonesia, Java	HQ697191	HQ697088
***Phoebe* (6)**				
*P. chekiangensis* C. B. Shang	Li J. & Li L. 20070188 (HITBC)	China, Zhejiang	FJ755407	HQ697128
*P. cuneata* (Blume) Blume	Arifiani 40 (MO)	Indonesia, Java	HQ697202	HQ697130
*P. formosana* (Hayata) Hayata	Rohwer 156 (MJG)	Germany, Bonn	HQ697205	HQ697136
*P. lanceolata* (Wall. ex Nees) Nees	Chen J. Q. et al. 2006093 (HITBC)	China, Guangdong	FJ755410	HQ697141
*P. nanmu* (Oliv.) Gamble	Chen J. Q. et al. 2005002 (HITBC)	China, Yunnan	FJ755409	HQ697150
*P. neurantha* (Hemsl.) Gamble	Li J. & Li L. 20070214 (HITBC)	China, Zhejiang	HQ697209	HQ697151
**Outgroups**				
***Actinodaphne* (1)**				
*A. trichocarpa* C. K. Allen	Li L. 20070282 (HITBC)	China, Sichuan	HQ697214	HQ697166
***Lindera* (1)**				
*L. erythrocarpa* Makino	Li J. & Li L. 20070203 (HITBC)	China, Zhejiang	HQ697215	HQ697167
***Litsea* (1)**				
*L. auriculata* Chien & Cheng	Li J. & Li L. 20070195 (HITBC)	China, Zhejiang	HQ697217	HQ697174
***Neolitsea* (1)**				
*N. howii* C. K. Allen	Li L. & Wang Z. H. 20070379 (HITBC)	China, Hainan	HQ697220	HQ697178

DNA sequences were aligned using Clustal X 2.1 (Larkin et al. 2007) and adjusted manually. The combined dataset including ITS and *LEAFY* intron II sequences for phylogenetic analysis was built according to the works of Li et al. (2011) and Mo et al. (2017a). Phylogenetic analyses were performed using the maximum parsimony (MP) and Bayesian inference (BI) methods.

The MP analysis was performed using PAUP* 4.0b10 (Swofford 2003). The heuristic search was performed with 1000 random sequence addition replicates, tree-bisection-reconnection (TBR) swapping, MulTrees on, and all characters equally weighted. Bootstrap values of the internal nodes were obtained with 1000 bootstrap replicates. The BI analysis was performed using MrBayes v.3.2.6 (Ronquist and Huelsenbeck 2003). Different DNA sequences were defined as separate data partitions. The evolutionary model for each partition (ITS: TVM+I+G; *LEAFY* intron II: HKY+G) was estimated using jModelTest v.2.1.10 (Darriba et al., 2012) with the Akaike information criterion (AIC) (Akaike 1974; Posada and Buckley 2004). The Markov chain Monte Carlo (MCMC) algorithm was run for 10 million generations with a sampling frequency of one every 1000 generations, and the first 25% trees were discarded as burn-in.

## Results

**Figure 1.**
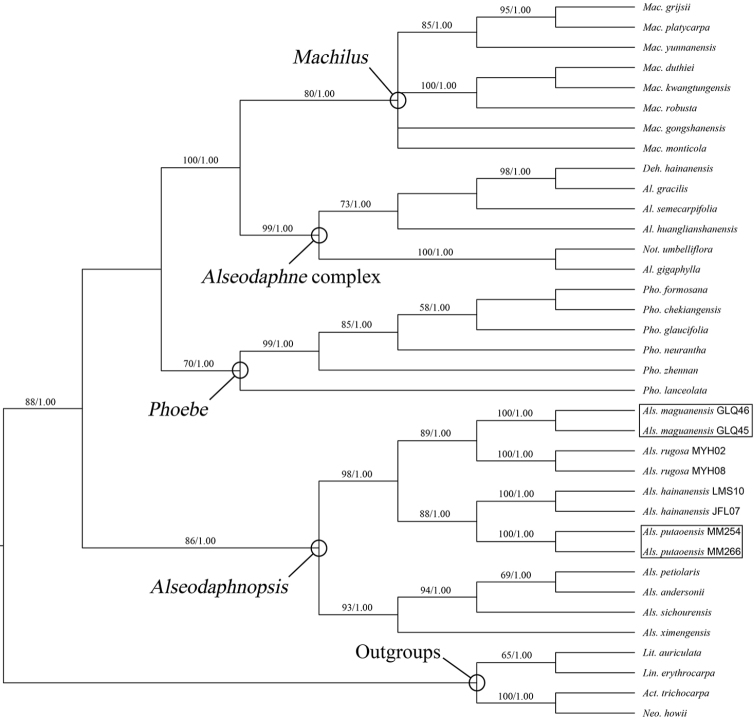
Bayesian consensus tree of ITS + *LEAFY* intron II combined dataset. MP bootstrap (BS ≥ 50%) and Bayesian posterior probability (PP ≥ 0.95) values are shown above branches. *Act.* = *Actinodaphne*, *Al.* = *Alseodaphne*, *Als.*= *Alseodaphnopsis*, *Deh.* = *Dehaasia*, *Lin.* = *Lindera*, *Lit.* = *Litsea*, *Mac.* = *Machilus*, *Neo.* = *Neolitsea*, *Not.* = *Nothaphoebe*, *Pho.* = *Phoebe*.

The MP and BI analyses of the ITS + *LEAFY* intron II combined dataset generated congruent topologies. The Bayesian consensus tree with MP bootstrap (BS) and Bayesian posterior probability (PP) values is shown in Fig. 1. All *Alseodaphnopsis* individuals investigated in the present study formed a monophyletic clade that receives a BS of 86% and a PP of 1.00. Within the *Alseodaphnopsis* clade, two well-supported subclades are found, each consisting of four species. The new species *Alseodaphnopsis maguanensis* is sister to *A. rugosa* (BS 89%, PP 1.00) and new species *A. putaoensis* is sister to *A. hainanensis* (BS 88%, PP 1.00).

### Taxonomic treatments

#### Alseodaphnopsis maguanensis

Taxon classificationPlantaeLauralesLauraceae

L.Li & J.Li sp. nov.

75F2471E-A750-56E8-891A-956A9D2B24E9

urn:lsid:ipni.org:names:77204194-1

Figs 2 , 3

##### Diagnosis.

*Alseodaphnopsis maguanensis* is morphologically similar and phylogenetically closely related to *A. rugosa*, but can be distinguished by its much larger fruit (4–5 × 5–6 cm vs. ca. 2.5 × 3 cm), mature fruit color (brown vs. deep purple or black) and different fruiting phenology.

##### Type.

China. Yunnan Province: Maguan County, Houcao, Gulinqing Provincial Natural Reserve, in tropical montane forest, 800 m a.s.l., 14 May 2016, flowering, Lang Li et al., *GLQ26* (holotype: HITBC!).

##### Description.

Trees evergreen, up to 20 m tall. Branchlets terete, 3–6 mm in diameter, grayish, glabrous, wrinkled, with lenticels and leaf scars. Terminal buds glabrous. Leaves clustered at apex of branchlet, alternate or subverticillate; petiole robust, 2–3 mm thick, 1.5–2.5 cm long, concave-convex; leaf blade green adaxially, glaucous abaxially when young but green or pale green when mature, oblong-obovate or oblong-oblanceolate, 12–32 × 3.5–9 cm, leathery, glabrous on both surfaces, midrib conspicuously elevated abaxially, impressed adaxially, lateral veins 8–12 pairs, veins and veinlets conspicuous, reticulate, elevated on both surfaces when dry, base cuneate, apex shortly acuminate. Panicles subterminal, clustered at apex of branchlet, 15–20 cm, many-flowered; peduncle 4.5–10 cm, glabrous. Pedicels slender, 5–8 mm, glabrous. Perianth lobes 6, glabrous outside, white pubescent inside, outer ones broadly ovate, ca. 2 × 1.5 mm, acute, inner ones broadly ovate, ca. 2.5 × 2 mm, acute, all deciduous when in fruit. Fertile stamens 9, ca. 2 mm in 1^st^ and 2^nd^ whorls, ca. 2.2 mm in 3^rd^ whorl; filaments villous, almost as long as anthers in 1^st^ and 2^nd^ whorls, slightly longer than anthers in 3^rd^ whorl, those of 3^rd^ whorl each with 2 shortly stalked orbicular-cordate glands, others glandless; anthers of 1^st^ and 2^nd^ whorls ovate, with 2 upper smaller cells and 2 lower large cells, cells all introrse, anthers of 3^rd^ whorl elliptic, with 4 extrorse cells. Staminodes conspicuous, ca. 1.5 mm, sagittate, stalked. Ovary ovoid, ca. 1.2 mm, glabrous, attenuate into a ca. 0.8 mm long style; stigma discoid, inconspicuous. Infructescence subterminal, 10–18 cm, robust, glabrous, with only one well-developed fruit. Fruit large, oblate, 4–5 × 5–6 cm, immature fruit green, brown when mature, fruit stalk robust, 3–4 mm in diameter, apex dilated, 5–10 mm in diameter, sometimes nearly cylindric, fleshy and warty when fresh.

**Figure 2.**
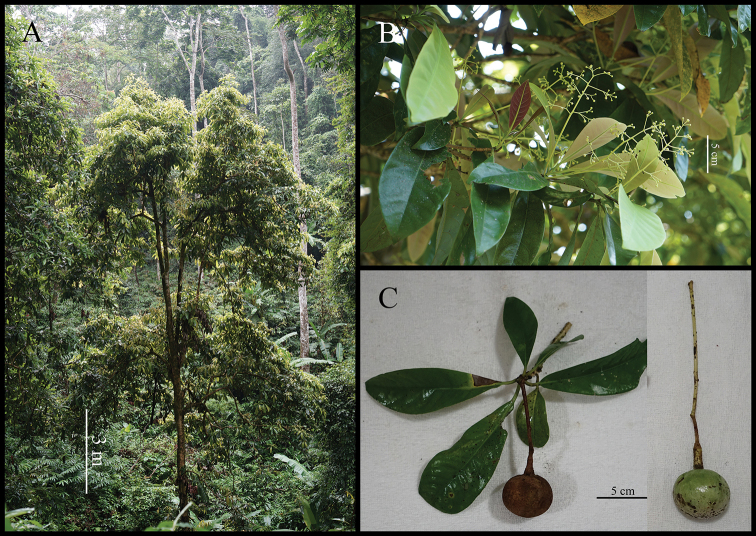
*Alseodaphnopsis maguanensis***A** habitat **B** branchlet with inflorescences **C** branchlet with mature fruit, immature fruit. Photographed by Lang Li.

**Figure 3.**
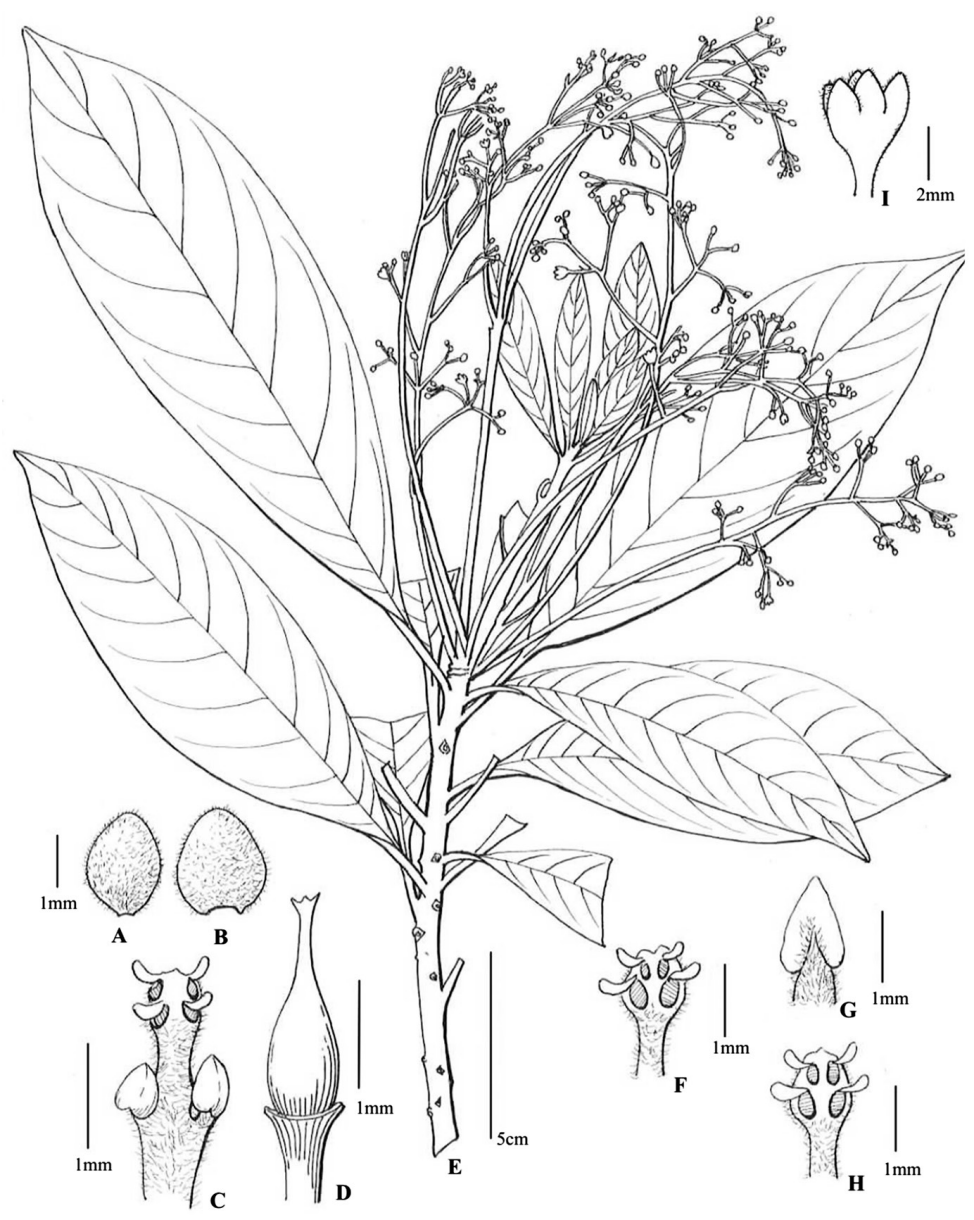
*Alseodaphnopsis maguanensis***A** outer perianth lobe (inside view) **B** inner perianth lobe (inside view) **C** third whorl stamen **D** pistil **E** flowering branch **F** second whorl stamen **G** staminode **H** first whorl stamen **I** flower (lateral view). Illustration by Ling Wang from Mo et al. (2017b).

##### Phenology.

Flowering from May to June and fruiting from July to September.

##### Etymology.

The species is named after the type locality, Maguan County, in Yunnan Province, China.

##### Distribution and habitat.

Currently known only from the type locality in Maguan, Yunnan Province, southwestern China. Tropical montane forests in valleys; ca. 800m.

##### Preliminary conservation status.

Currently, *A. maguanensis* is only known from Maguan (Yunnan Province, China) with two populations, which are all located in a small natural reserve (ca. 71 km^2^), each population with less than 50 mature individuals (seedlings can be found near the mature individuals), and no other occurrence in adjacent regions of SE Yunnan and N Vietnam. Thus, the preliminary conservation status for *A. maguanensis* is suggested as critically endangered (CR C2a(i)) according to the IUCN Red List Categories and Criteria (IUCN 2012).

##### Additional specimen examined (paratype).

China. Yunnan Province: Maguan County, Shangba, Gulinqing Provincial Natural Reserve, in tropical montane forest, 800 m a.s.l., 28 August 2016, fruiting, Lang Li et al., *2016033* (HITBC!).

#### Alseodaphnopsis putaoensis

Taxon classificationPlantaeLauralesLauraceae

L.Li, Y.H.Tan & J.Li sp. nov.

6101EB74-3CB3-5E3E-9C96-123DB06F3D27

urn:lsid:ipni.org:names:77204195-1

Fig. 4

##### Diagnosis.

*Alseodaphnopsis putaoensis* is morphologically similar to *A. rugosa*, but phylogenetically closely related to *A. hainanensis*. It can be distinguished from them by its fruit stalk characters (apex slightly dilated, not fleshy, red and warty when fresh vs. apex dilated, nearly cylindric, fleshy, red and warty when fresh), much larger fruit (6–6.5 × 7–10 cm vs. ca. 2.5 × 3 cm and 1.2–2 cm), mature fruit color (brown vs. deep purple or black) and different fruiting phenology.

##### Type.

Myanmar. Kachin State: Putao County, on the way from Masabu to Namti, in tropical montane forest, 1000 m a.s.l., 13 May 2017, fruiting, Lang Li & Hui Ma *MM271* (holotype: HITBC!).

##### Description.

Trees evergreen, up to 15 m tall. Branchlets terete, robust, 7–10 mm in diameter, brownish or dark brown, glabrous, wrinkled, with lenticels and leaf scars. Terminal buds glabrous. Leaves clustered at apex of branchlet, subverticillate; petiole robust, 2–3 mm thick, 2.5–4.5 cm long, concave-convex; leaf blade green adaxially, glaucous abaxially, oblong-obovate or oblong-oblanceolate, 18–35 × 6–9 cm, leathery, glabrous on both surfaces, midrib conspicuously elevated abaxially, impressed adaxially, lateral veins 8–12 pairs, veins and veinlets conspicuous, reticulate, elevated on both surfaces when dry, base cuneate, apex shortly acuminate. Flowers unknown. Infructescence subterminal, 8–10 cm, robust, glabrous, with only one well-developed fruit. Fruit large, oblate, 6–6.5 × 7–10 cm, immature fruit green, brown when mature, fruit stalk robust, 3–4 mm in diameter, apex slightly dilated, 5–6 mm in diameter.

**Figure 4.**
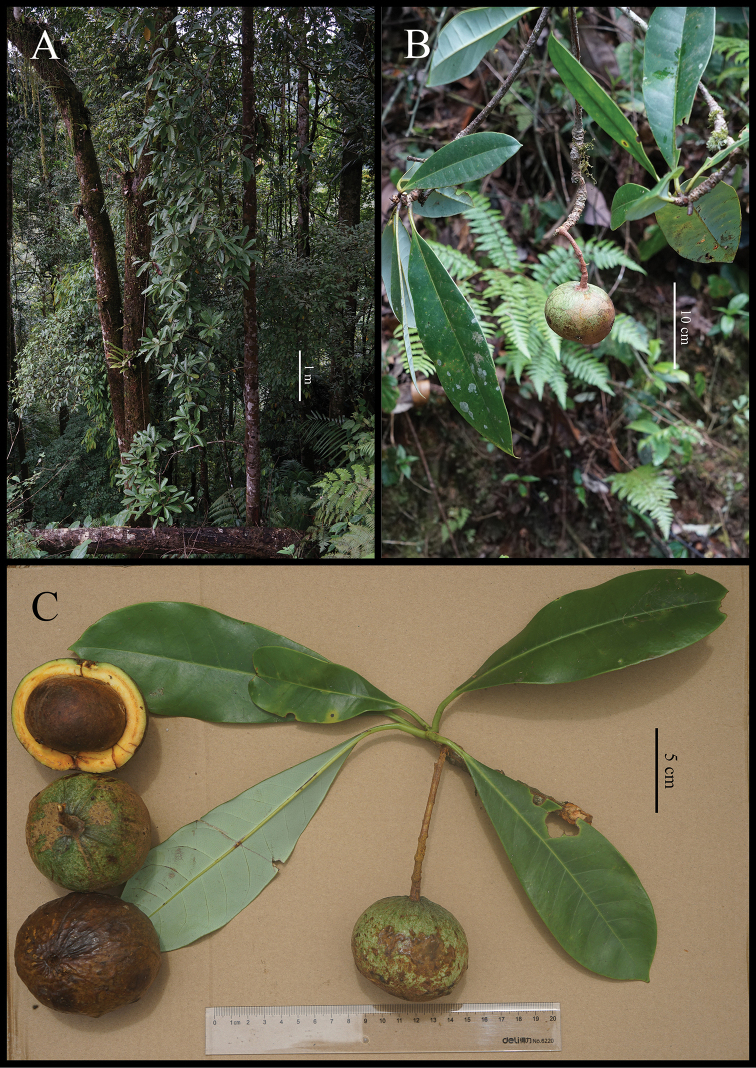
*Alseodaphnopsis putaoensis***A** habitat **B** branchlet with immature fruit **C** branchlet with immature fruit, immature and mature fruits. Photographed by Lang Li.

##### Phenology.

Individuals with immature or mature fruits have been collected in May, fruiting may be from April to June.

##### Etymology.

The species is named after the type locality, Putao County, in Kachin State, Myanmar.

##### Distribution and habitat.

Currently known only from the type locality in Putao, Kachin State, northern Myanmar. Tropical montane forests on mountain slopes or in valleys; 600–1400m.

##### Preliminary conservation status.

During the field survey in Putao (Kachin State, Myanmar), several populations of *A. putaoensis* were found and at least two of them with more than 50 mature individuals (seedlings could be found near the mature individuals) each. In future field surveys, potential populations and more individuals are expected to be found in Putao and adjacent regions. Currently, some localities of *A. putaoensis* have not been legally protected. The habitat fragmentation, as well as ongoing road construction and continuous logging, are threatening its survival. Thus, the preliminary conservation status for *A. putaoensis* is suggested as vulnerable (VU C12a(i)) according to the IUCN Red List Categories and Criteria (IUCN 2012).

##### Additional specimen examined (paratype).

Myanmar. Kachin State: Putao County, on the way from Masabu to Namti, in tropical montane forest, 900 m a.s.l., 13 May 2017, fruiting, Lang Li & Hui Ma *MM254* (HITBC!).

## Discussion

The close relationships of *Alseodaphnopsis maguanensis*, *A. rugosa*, *A. putaoensis* and *A. hainanensis* were indicated by the phylogenetic analyses. They formed a well-supported subclade within the *Alseodaphnopsis* clade, and *A. maguanensis* is sister to *A. rugosa* while *A. putaoensis* is sister to *A. hainanensis* (Fig. 1). *Alseodaphnopsis maguanensis*, *A. putaoensis* and *A. rugosa* are very similar in their vegetative characters (e.g. leaf and branchlet characters), but *A. hainanensis* can be easily distinguished from them by its narrowly elliptic and smaller leaves (vs. oblong-obovate or oblong-oblanceolate and larger leaves of *A. maguanensis*, *A. putaoensis* and *A. rugosa*) (Li et al. 2008). The fruit stalk and fruit characters are very important to distinguish *Alseodaphnopsis maguanensis*, *A. putaoensis* and *A. rugosa*. *Alseodaphnopsis putaoensis* has the largest fruit (6–6.5 × 7–10 cm, brown when mature) with the fruit stalk slightly dilated at the top (not fleshy and warty when fresh), while *A. maguanensis* and *A. rugosa* have relative smaller fruits (4–5 × 5–6 cm, brown when mature and ca. 2.5 × 3 cm, deep purple or black when mature) with the fruit stalks distinctly dilated at the top (nearly cylindric, fleshy and warty when fresh).

*Alseodaphnopsis maguanensis*, *A. rugosa*, *A. putaoensis* and *A. hainanensis* also have different fruiting phenologies. *Alseodaphnopsis maguanensis* is fruiting from July to September, both immature and mature fruits can be found in August. According to the work of Li et al. (2008), *Alseodaphnopsis rugosa* is fruiting from July to December. However, based on specimen and field observations, very few individuals with young fruits were collected in August, most fruiting individuals were collected from October to December. For *Alseodaphnopsis putaoensis*, individuals with immature or mature fruits have been collected in May, fruiting may be from April to June. *Alseodaphnopsis hainanensis* is fruiting from October to February (Li et al. 2008). A detailed comparison of the morphological differences among these four taxa, as well as their phenologies and distributions, is given in Table 2. Unfortunately, the flowers of *Alseodaphnopsis putaoensis* and *A. rugosa* remain unknown. The evidence from both phylogenetic and morphological analyses support the recognition of *Alseodaphnopsis maguanensis* and *A. putaoensis* as distinct species in the genus.

**Table 2.** Comparison of key morphological characters, phenologies and distributions of *Alseodaphnopsis maguanensis*, *A. rugosa*, *A. putaoensis* and *A. hainanensis*.

Morphological characters	*Alseodaphnopsis maguanensis*	*Alseodaphnopsis rugosa*	*Alseodaphnopsis putaoensis*	*Alseodaphnopsis hainanensis*
Leaf blade	oblong-obovate or oblong-oblanceolate, 12–32 × 3.5–9 cm, green adaxially, glaucous abaxially when young but green or pale green when mature	oblong-obovate or oblong-oblanceolate, 15–36 × 4–10 cm, green adaxially, glaucous abaxially	oblong-obovate or oblong-oblanceolate, 18–33 × 6–9 cm, green adaxially, glaucous abaxially	narrowly elliptic, 6–16 × 1.5–4.2 cm, green adaxially, glaucous abaxially when young but green or pale green when mature
Infructescence	with only one well-developed fruit	with one or several well-developed fruits	with only one well-developed fruit	with one or several well-developed fruits
Fruit stalk	apex dilated, 5–10 mm in diam., sometimes nearly cylindric, fleshy and warty when fresh	apex dilated, nearly cylindric, 5–8 mm in diam., fleshy, red and warty when fresh	apex slightly dilated, 5–6 mm in diam.	apex dilated, nearly cylindric, 5–8 mm in diam., fleshy, red and warty when fresh
Fruit	oblate, 4–5 × 5–6 cm, brown when mature	oblate, ca. 2.5 × 3 cm, deep purple or black when mature	oblate, 6–6.5 × 7–10 cm, brown when mature	globose or ovoid, 1.2–2 cm, deep purple or black when mature
**Flowering Phenology**	May–Jun	–	–	Jul
**Fruiting Phenology**	Jul–Sep	Jul–Dec (Fruits mostly found from Oct to Dec)	Apr–Jun	Oct–Feb of next year
**Distribution**	SW China (Yunnan)	S China (Hainan)	N Myanmar (Kachin)	S China (Hainan), N Vietnam (Lào Cai)

With *Alseodaphnopsis maguanensis*, *A. putaoensis* and the recently described *A. ximengensis* H. W. Li & J. Li from Ximeng, Yunnan Province, China (Mo et al. 2017a) included, *Alseodaphnopsis* currently has only 11 recorded species, indicating that the species diversity of the genus is still in need of investigation and open to discovery. More new species of *Alseodaphnopsis* are expected to be discovered in the northern marginal zone of the Asian tropics when more field investigations are conducted in this region.

## Supplementary Material

XML Treatment for Alseodaphnopsis maguanensis

XML Treatment for Alseodaphnopsis putaoensis
